# Genetic characterization and whole-genome sequencing-based genetic analysis of influenza virus in Jining City during 2021–2022

**DOI:** 10.3389/fmicb.2023.1196451

**Published:** 2023-06-22

**Authors:** Libo Li, Tiantian Liu, Qingchuan Wang, Yi Ding, Yajuan Jiang, Zengding Wu, Xiaoyu Wang, Huixin Dou, Yongjian Jia, Boyan Jiao

**Affiliations:** ^1^Department of Laboratory, Jining Center for Disease Control and Prevention, Jining, China; ^2^Department of Medicine, Jining Municipal Government Hospital, Jining, China; ^3^Department of AI and Bioinformatics, Nanjing Chengshi BioTech (TheraRNA) Co., Ltd., Nanjing, China

**Keywords:** influenza virus, whole-genome sequencing, mutation, genome characterization, Jining City

## Abstract

**Background:**

The influenza virus poses a significant threat to global public health due to its high mutation rate. Continuous surveillance, development of new vaccines, and public health measures are crucial in managing and mitigating the impact of influenza outbreaks.

**Methods:**

Nasal swabs were collected from individuals with influenza-like symptoms in Jining City during 2021-2022. Reverse transcription-quantitative polymerase chain reaction (RT-qPCR) was used to detect influenza A viruses, followed by isolation using MDCK cells. Additionally, nucleic acid detection was performed to identify influenza A H1N1, seasonal H3N2, B/Victoria, and B/Yamagata strains. Whole-genome sequencing was conducted on 24 influenza virus strains, and subsequent analyses included characterization, phylogenetic construction, mutation analysis, and assessment of nucleotide diversity.

**Results:**

A total of 1,543 throat swab samples were collected. The study revealed the dominance of the B/Victoria influenza virus in Jining during 2021-2022. Whole-genome sequencing showed co-prevalence of B/Victoria influenza viruses in the branches of Victoria clade 1A.3a.1 and Victoria clade 1A.3a.2, with a higher incidence observed in winter and spring. Comparative analysis demonstrated lower similarity in the HA, MP, and PB2 gene segments of the 24 sequenced influenza virus strains compared to the Northern Hemisphere vaccine strain B/Washington/02/2019. Mutations were identified in all antigenic epitopes of the HA protein at R133G, N150K, and N197D, and the 17-sequence antigenic epitopes exhibited more than 4 amino acid variation sites, resulting in antigenic drift. Moreover, one sequence had a D197N mutation in the NA protein, while seven sequences had a K338R mutation in the PA protein.

**Conclusion:**

This study highlights the predominant presence of B/Victoria influenza strain in Jining from 2021 to 2022. The analysis also identified amino acid site variations in the antigenic epitopes, contributing to antigenic drift.

## 1. Introduction

The influenza virus is a common pathogen in the human respiratory tract, causing approximately 290,000 to 650,000 deaths each year. It is considered one of the major public health problems worldwide (Chavez and Hai, 2021; Tyrrell et al., 2021). Influenza virus is a single positive-stranded RNA virus, which is further divided into four types: type A, type B, type C, and type D based on the antigenicity of nucleoprotein and matrix protein (Carascal et al., 2022). Influenza A and B viruses are the primary cause of illness in humans. Influenza A viruses are divided into subtypes based on the antigenicity of hemagglutinin (HA) and neuraminidase (NA). HA is divided into 18 subtypes, while NA is divided into 11 subtypes (Duraes-Carvalho and Salemi, 2018). In contrast, the influenza B virus has only one subtype, which is further divided into two lineages: Victoria lineage and Yamagata lineage based on the antigenic characteristics and HA sequence of influenza B (Hay et al., 2001; Toure et al., 2022).

Both influenza A and B viruses are composed of eight segments of the gene, including haemagglutinin (HA), neuraminidase (NA), matrix protein (MP), nucleoprotein (NP), nonstructural (NS), polymerase acidic (PA), polymerase basic 1 (PB1), and polymerase basic 2 (PB2) genes. These gene segments between different types can undergo genetic recombination (Zhu et al., 2013; Liang et al., 2022). Among these segments, the HA protein of influenza virus is a major surface antigen prone to mutation, which can cause antigenic changes that help the virus evade host immunity and cause influenza outbreaks (Liu et al., 2018; Wu and Wilson, 2020). Currently, the seasonal influenza virus pathogens that infect humans are mainly influenza A H1N1 influenza virus, seasonal influenza A H3N2 subtype virus, B Victoria series influenza virus, and B Yamagata series influenza virus (Wu and Wilson, 2020). These four influenza viruses alternate in prevalence during different years (Liu et al., 2022).

The influenza virus can cause fever, cough, sore throat, pneumonia, and even death, and the general population is susceptible. Vaccination is an effective method to prevent influenza. However, the influenza virus is prone to mutation, undergoes antigenic drift, and produces immune escape. Both NA and PA inhibitors are specific drugs for treating influenza; however, with the continuous use of drugs, the virus may develop drug-resistant mutations, which reduces the therapeutic effectiveness of the drugs.

Influenza is highly prevalent during the winter and spring months in northern China, and monitoring typically occurs from April to March of the following year (Liu et al., 2022). Jining, being one of the most densely populated cities in northern China, the threat of seasonal flu outbreaks in this region should not be underestimated. In this study, we analyzed the epidemic patterns and genome-wide characteristics of the influenza virus monitored in Jining City from 2021 to 2022. Our goal was to gain insight into the epidemic characteristics of the influenza virus and the evolutionary trends of circulating strains in order to improve outbreak prevention strategies.

## 2. Materials and methods

### 2.1. Specimens collection

According to the requirements of the National Influenza Center of China, between 1 April 2021 and 31 March 2022, throat swab specimens for influenza-like illness (ILI) (body temperature ≥ 38°C, with either cough or sore throat) were collected at Jining First People’s Hospital and Rencheng District Maternal and Child Health Planning Service Center. A minimum of 10 samples were collected each week from April 2021 to September 2021, and a minimum of 20 samples were collected each week from October 2021 to March 2022, resulting in a total of 1,543 samples. The collected specimens were immediately stored at 2–8°C and transferred to the Influenza Surveillance Network Laboratory of Jining Center for Disease Control and Prevention within 24 h for testing.

### 2.2. Laboratory testing

A volume of 200 μl throat swab sample was taken, and the automatic nucleic acid extraction instrument (GeneRotex 96) of Xi’an Tianlong Technology Co., Ltd. and the virus nucleic acid extraction reagent (T138) of Xi’an Tianlong Technology Co., Ltd. for nucleic acid extraction were used. Also, Guangzhou Daan Biotechnology Co., Ltd. Type A H1N1 (DS0042), Seasonal H3N2 (DS0091), B/Victoria series (D0970), and B/Yamagata series (D0960) influenza virus nucleic acid detection kit (PCR-fluorescent probe method) were used for nucleic acid detection.

### 2.3. Influenza virus culture

Madin–Darby canine kidney (MDCK) cells were cultured in DMEM (GIBCO 11995–065) medium with 10% FBS (GIBCO 16000–044) at 37°C. After the cells reached 90% confluence, the culture medium was discarded and washed with 5-ml PBS for three times. Then, 1 ml of a throat swab sample with positive influenza virus was inoculated; incubated at 35°C for 1 h; discarded the throat swab sample; added 5 mL of DMEM (containing 2 μg/mL TPCK-trypsin, 100 U/ml penicillin, and 100 μg/ml chain tetracycline), and incubated at 35°C. After 3–4 days, the cells were frozen–thawed three times, and 1% red blood cells were used to measure the HA titer of influenza virus strains. Twenty-four influenza virus strains with virus titer ≥1:8 was isolated, and the strain numbers were as follows: B/shandongrencheng/11484, 11,485, 11,486, 11,487, 11,488, 11,494, 11,495, 11,499, 11,504, 11,506/2021; B/shandongrencheng/1115, 1,122, 1,125, 1,126, 1,127, 1,128, 1,169, 1,176, 1,210, 1,211, 1,252, 1,291, 1,354, 1,356/2022. Sampling dates for the 24 strains were 5 November 2021 (5 strains), 5 December 2021 (5 strains), 6 January 2022 (6 strains), 5 February 2022 (5 strains), and 3 March 2022 (3 strains). Of the 24 strains, 16 strains were isolated from the male patients and 8 strains were isolated from the female patients. Age distribution was as follows: 1 patient aged 0–5 years, 8 patients aged 6–15 years, 6 patients aged 16–25 years, 8 patients aged 26–60 years, and 1 patient aged above 61 years.

### 2.4. Whole-genome sequencing

Influenza virus gene capture using Beijing Micro Future’s ULSENTM^®^ Ultra-Sensitive Influenza Virus Whole-Genome Capture Kit. Reaction system: DEPC-treated ddH_2_O, 12 μl; B whole-genome amplification mix, 25 μl; BWGP-Mix, 4 μl; EF Enzyme 1 μl; and virus culture cell line nucleic acid, 8 μl. Reaction conditions: 45°C, 60 min; 55°C, 30 min; 94°C, 2 min; 94°C, 20 s; 40°C, 30 s; 68°C, 3 min 30 s; 5 cycles; 94°C, 20 s; 58°C, 30 s; 68°C, 3 min 30 s; 40 cycles; and 68°C, 10 min. After purifying the product with AMPure XP (BECKMAN A63880), the library was amplified with Nextera® XT Library Prep Kit (Illumina 15032352) and sequenced with Illumina NextSeq2000 sequencer and NextSeqTM 2000 P3 Reagent Cartridge 300 cycles (Illumina 20045959) sequencing reagent.

### 2.5. Genome assembly and analysis

Whole genome of the influenza virus on sequenced data was assembled using the QIAGEN CLC Genomics Workbench 21 software. Download 2021–2022 Northern Hemisphere BV influenza virus vaccine representative strain B/Washington/02/2019 (EPI_ISL_341131), 2021–2022 global BV influenza virus sequence, and BV early isolate sequence from the GISAID database. Using the Kimura two-parameter model of the Neighbor-joining method of the MEGA 7.0.14 software to construct the evolutionary tree of eight gene segments of the influenza virus. Nucleotide and amino acid homology analysis was performed using MegAlign of the DNASTAR 7.0.1 software; the amino acid sequence alignment was performed using the align of the MEGA 7.0.14 software to analyze the amino acid difference sites; and analysis of protein *N*-glycosylation sites was carried out using the NetNGlyc 1.0 Server software. The data presented in the study are deposited in the e Global Initiative on Sharing All Influenza Data (GISAID)^1^ repository, accession number list in Supplementary Table S1.

## 3. Results

### 3.1. Positive rate of BV influenza virus

From April 2021 to March 2022, 1,543 specimens from patients with influenza-like illness (ILI) were tested, of which 380 (24.63%) were positive for BV influenza virus. No cases of influenza A H1N1, seasonal H3N2, and BY influenza viruses were detected. The BV influenza viruses were not detected from April 2022 to September 2022, but began to be detected in ILI in late October 2022. The highest positive rate of influenza viruses in ILI was observed in December 2022 and January 2022. The favorable rates of BV influenza viruses in different months were statistically significant (*χ*^2^ = 409.002, *p* < 0.001). The favorable rates of BV influenza virus in males and females were 22.67 and 26.69%, respectively (*χ*^2^ = 3.382, *p* = 0.07). The positive rates of BV influenza virus in different age groups were 8.15% for 0–5 years; 32.22% for 6–15 years; 26.91% for 16–25 years, 33.98% for 26–60 years, and 9.46% for those above 60 years, respectively (*χ*^2^ = 103.411, *p* < 0.001) (Table 1). In summary, the study found a high prevalence of BV influenza virus in ILI cases in Jining City from October 2021 to March 2022, with the highest positive rate observed in December 2021 and January 2022. The virus showed significant differences in favorable rates across different age groups and months.

**Table 1:** The positive rate of nucleic acid testing and composition characteristics of BV influenza in Jining between 2021–2022.

Variables	Testing	Influenza A (H1N1), Influenza A (H3N2), Influenza B (Yamagata)		Influenza B (Victoria)	
	Specimens number	Positives number	Positive rate (%)	Positives number	Positive rate (%)
Month
April–September 2021	309	0	0	0	0
October 2021	171	0	0	3	1.75
November 2021	204	0	0	41	20.10
December 2021	232	0	0	129	56.60
January 2022	242	0	0	133	54.96
February 2022	180	0	0	49	27.22
March 2022	205	0	0	25	12.20
Gender
Male	790	0	0	179	22.67
Female	753	0	0	201	26.69
Age (in years)
0–5	405	0	0	33	8.15
6–15	329	0	0	106	32.22
16–25	223	0	0	60	26.91
26–60	512	0	0	174	33.98
≥61	74	0	0	7	9.46
**Total**	1543	0	0	380	24.63

### 3.2. Nucleotide diversity of B/Victoria influenza viruses

This study successfully sequenced 24 whole genomes of B/Victoria influenza viruses. The nucleotide similarity of 8 gene fragments in the sequences of 24 strains ranged from 98.3 to 100%. Compared with the WHO-recommended BV influenza virus vaccine strain B/Washington/02/2019 for the Northern Hemisphere 2021–2022, the nucleotide similarity of the 8 gene segments varied from 98.2 to 99.7%. The amino acid similarity of the encoded protein was the lowest at 97.2% and was the highest at 100% (Table 2).

**Table 2:** Similarity analysis of the whole genome of BV influenza virus in Jining.

Gene	Protein	Sequence similarity between 24 strains (%)		Similarity compared to B/Washington/02/2019(%)	
		Nucleotides	Amino acid	Nucleotides	Amino acid
HA	HA	98.3–100	98.2–100	98.3–99.0	97.9–98.8
NA	NA	98.6–100	97.6–100	98.8–99.3	98.7–99.4
MP	M1	98.7–100	99.2–100	98.2–98.7	98.8–99.6
	M2		96.3–100		97.2–99.1
NP	NP	98.8–100	99.1–100	99.1–99.6	99.1–100
NS	NS1	99.0–100	97.5–100	98.8–99.2	98.2–99.6
	NEP		99.2–100		97.5–98.4
PA	PA	98.6–100	98.3–100	98.7–99.4	98.6–99.7
PB1	PB1	98.8–100	98.8–100	99.2–99.7	99.2–99.7
PB2	PB2	98.8–100	98.8–100	98.2–98.6	99.1–99.9

Additionally, the evolutionary distances of HA, NA, MP, NP, NS, PA, PB1, and PB2 genes in the 24 sequences were 0.0076, 0.0078, 0.0069, 0.0056, 0.0045, 0.0052, 0.0048, and 0.0065, respectively, when compared with B/Washington/02/2019. The evolutionary distances of HA, NA, MP, NP, NS, PA, PB1, and PB2 genes were 0.0129, 0.0093, 0.0159, 0.0063, 0.0090, 0.0078, 0.0048, and 0.0159, respectively (Table 2). Overall, this study provides valuable insights into the genetic characteristics and evolutionary distances of B/Victoria influenza viruses, which can help inform the development and selection of appropriate vaccine strains.

### 3.3. Analysis of genetic variation and evolution of B/Victoria influenza viruses

**Figure 1:**
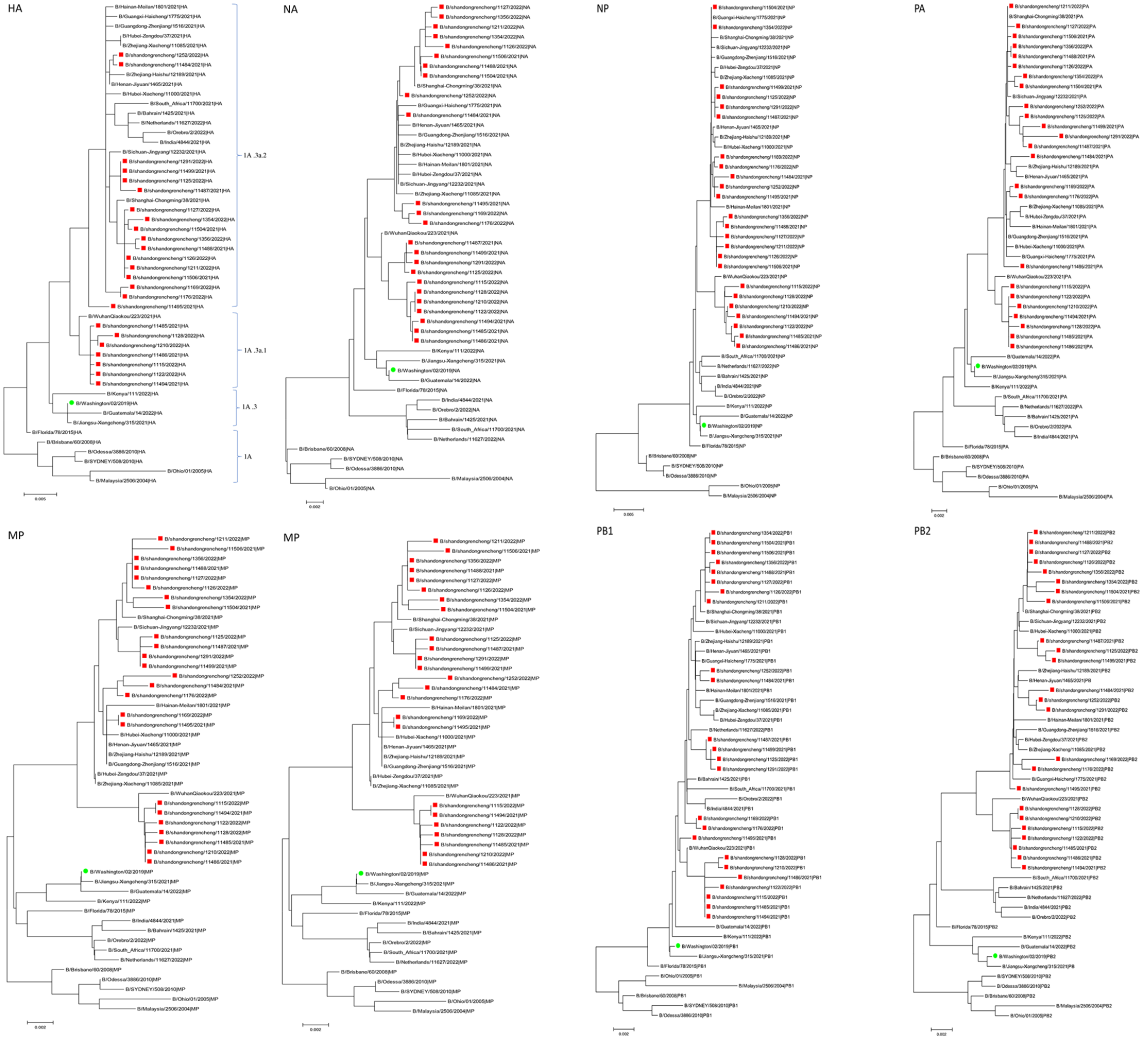
The phylogenetic analysis of influenza viruses circulating in Jining during 2021–2022 (red) compared with the Northern Hemisphere vaccine strain B/Washington/02/2019 (green). HA, hemagglutinin. NA, neuraminidase.

In this study, the genetic evolution of B/Victoria influenza viruses in Jining was analyzed using the sequences of their eight segments. A phylogenetic tree was constructed using the eight-segment sequences from 24 B/Victoria strains, including the global BV influenza virus (2021–2022), influenza vaccine strains recommended by WHO, and early BV isolates. The results revealed that the eight segments of the seven sequences were all closely related and located in the same evolutionary clade. Notably, the HA genes of these sequences all belonged to the Victoria clade 1A.3a.1. The remaining strains were found in a separate evolutionary clade, with their HA genes belonging to the Victoria clade 1A.3a.2 (Figure 1). Overall, the phylogenetic analysis of the eight-segment sequences of B/Victoria influenza viruses in Jining revealed two distinct evolutionary clades, with the HA genes of the sequences belonging to either the Victoria clade 1A.3a.1 or the Victoria clade 1A.3a.2.

### 3.4. Amino acid variant analysis

Compared with B/Washington/02/2019, the HA, NA, and PA genes showed the highest number of amino acid variation sites. HA is crucial for the antigenic variation of the influenza virus, and its heavy chain region contains both antigenic determinants and receptor binding sites (Liu et al., 2018). These include the 120-loop (116–137 aa), 150-loop (141–150 aa), 160-loop (160–172 aa), and 190-helix (193–202 aa) for antigenic epitopes, as well as the 140-loop (136–143 aa), 190-helix (193–202 aa), and 240-loop (237–242 aa) for the HA receptor binding site (Wang et al., 2007; Liu et al., 2018). Table 3 shows that out of the 24 HA protein sequences, 20 variations were found, with 9 occurring in epitopes, including 5 in the 120-loop, 2 in the 150-loop, 1 in the 160-loop, and 1 in the 190-helix. Additionally, there were three mutations in the HA receptor binding site, with 1 in the 190-helix and 2 in the 240-loop.

**Table 3:** Analysis of amino acid variation sites of B/Victoria influenza viruses in Jining City during 2021–2022.

Virus strain	**HA**																				**NA**																				**M1**					
	58	59	117	122	127	129	133	144	150	169	184	197	203	217	220	238	241	279	411	559	12	35	45	51	53	59	73	128	148	193	197	219	233	303	336	343	371	390	399	453	15	42	46	89	136	245
B/Washington/02/2019	L	N	V	H	A	D	R	P	N	A	G	N	K	A	V	G	P	R	Q	V	F	D	I	P	D	N	L	K	G	V	D	N	G	V	P	K	K	D	V	G	T	A	I	T	G	K
B/shandongrencheng/11484/2021	L	N	V	Q	T	D	G	L	K	A	E	D	R	A	V	G	P	K	Q	V	F	D	I	P	N	S	L	K	G	V	D	N	E	V	P	K	K	D	V	G	I	A	I	R	G	K
B/shandongrencheng/11485/2021	L	N	V	H	A	D	G	P	K	A	E	D	K	A	M	G	Q	K	Q	V	F	G	I	Q	D	N	L	K	G	V	D	N	G	I	P	E	K	D	V	R	I	A	I	R	G	R
B/shandongrencheng/11486/2021	L	N	V	H	A	D	G	P	K	A	E	D	K	A	M	G	Q	K	Q	I	F	G	I	Q	D	N	L	K	G	V	D	N	G	I	P	E	K	D	V	R	I	A	I	R	G	K
B/shandongrencheng/11487/2021	H	K	V	Q	T	D	G	L	K	A	E	D	R	A	V	G	P	K	Q	V	F	D	I	P	D	N	L	K	G	V	D	N	G	I	P	E	K	E	V	G	I	A	V	R	G	K
B/shandongrencheng/11488/2021	L	N	I	Q	T	D	G	L	K	T	E	D	R	A	V	G	P	K	Q	V	F	D	I	P	N	S	L	K	G	V	D	N	E	V	P	E	K	D	V	G	I	A	I	R	G	K
B/shandongrencheng/11494/2021	L	N	V	H	A	D	G	P	K	A	E	D	K	A	M	G	Q	K	Q	V	F	G	I	Q	D	N	L	K	G	V	D	N	G	I	P	E	K	D	A	R	I	A	I	R	G	K
B/shandongrencheng/11495/2021	L	N	V	Q	T	D	G	P	K	A	E	D	K	A	V	G	Q	K	Q	V	F	D	I	P	N	S	F	K	G	V	D	N	E	I	P	K	N	D	V	G	I	A	I	T	G	K
B/shandongrencheng/11499/2021	L	N	V	Q	T	D	G	L	K	A	E	D	R	A	V	G	P	K	Q	V	F	D	I	P	D	N	L	K	G	V	D	N	G	I	P	E	K	E	V	G	I	A	I	R	G	K
B/shandongrencheng/11504/2021	L	N	V	Q	T	D	G	L	K	T	E	D	R	A	V	G	P	K	Q	V	F	D	I	P	N	S	L	K	G	V	D	N	E	V	P	E	K	D	V	G	I	A	I	R	G	K
B/shandongrencheng/11506/2021	L	N	V	Q	T	D	G	L	K	T	E	D	R	A	V	G	P	K	Q	V	F	D	I	P	N	S	L	R	G	V	D	N	E	V	P	E	K	D	V	G	I	A	I	R	G	K
B/shandongrencheng/1115/2022	L	N	V	H	A	D	G	P	K	A	E	D	K	A	M	G	Q	K	Q	V	F	G	I	Q	D	N	L	K	G	V	D	N	G	I	P	E	K	D	V	G	I	A	I	R	G	K
B/shandongrencheng/1122/2022	L	N	V	H	A	D	G	P	K	A	E	D	K	V	M	G	Q	K	Q	V	F	G	I	Q	D	N	L	K	G	V	D	N	G	I	P	E	K	D	V	R	I	A	I	R	G	K
B/shandongrencheng/1125/2022	L	N	V	Q	T	D	G	L	K	A	E	D	R	A	V	G	P	K	Q	V	F	D	I	P	D	N	L	K	G	V	D	N	G	I	P	E	K	E	V	G	I	A	I	R	G	K
B/shandongrencheng/1126/2022	L	N	V	Q	T	D	G	L	K	T	E	D	R	A	V	G	P	K	Q	V	F	D	M	P	N	S	L	K	R	V	D	N	E	V	P	E	K	D	V	G	I	A	I	R	G	K
B/shandongrencheng/1127/2022	L	N	V	Q	T	D	G	L	K	T	E	D	R	A	V	G	P	K	Q	V	F	D	I	P	N	S	L	K	G	V	N	N	E	V	P	E	K	D	V	G	I	A	I	R	G	K
B/shandongrencheng/1128/2022	L	N	V	H	A	D	G	P	K	A	E	D	K	A	M	G	Q	K	Q	V	F	G	I	Q	D	N	L	K	G	V	D	N	G	I	P	E	K	D	V	R	I	V	I	R	G	K
B/shandongrencheng/1169/2022	L	N	V	Q	T	D	G	L	K	A	E	D	R	A	V	G	P	K	Q	V	F	D	I	P	N	N	L	K	G	V	D	N	E	V	P	K	N	D	V	G	I	A	I	T	G	K
B/shandongrencheng/1176/2022	L	N	V	Q	T	D	G	L	K	A	E	D	R	A	V	G	P	K	Q	V	F	D	I	P	N	S	L	K	G	V	D	S	E	V	P	K	N	D	V	G	I	A	I	R	G	K
B/shandongrencheng/1210/2022	L	N	V	H	A	D	G	P	K	A	E	D	K	A	M	G	Q	K	Q	V	F	G	I	Q	D	N	L	K	G	V	D	N	G	I	P	E	K	D	V	R	I	A	I	R	G	K
B/shandongrencheng/1211/2022	L	N	V	Q	T	D	G	L	K	T	E	D	R	A	V	G	P	K	Q	V	V	D	I	P	N	S	L	K	G	V	D	N	E	V	P	E	K	D	V	G	I	A	I	R	G	K
B/shandongrencheng/1252/2022	L	N	V	Q	T	D	G	L	K	A	E	D	R	A	V	G	P	K	Q	V	F	D	I	P	N	S	L	K	G	V	D	N	E	V	P	Q	K	D	V	G	I	A	I	R	E	K
B/shandongrencheng/1291/2022	L	N	V	Q	T	D	G	L	K	A	E	D	R	A	V	G	P	K	Q	V	F	D	I	P	D	N	L	K	G	V	D	N	G	I	P	E	K	E	V	G	I	A	I	R	G	K
B/shandongrencheng/1354/2022	L	N	V	Q	T	N	G	L	K	T	E	D	R	A	V	G	P	K	K	V	F	D	I	P	N	S	L	K	G	I	D	N	E	V	P	E	K	D	V	G	I	A	I	T	G	K
B/shandongrencheng/1356/2022	L	N	V	Q	T	D	G	L	K	T	E	D	R	A	V	E	P	K	Q	V	F	D	I	P	N	S	L	K	G	V	D	N	E	V	T	E	K	D	V	G	I	A	I	R	G	K

Virus strain	M2						NP									NS1														NEP		PA														
	14	24	70	73	79	88	37	51	78	113	120	128	183	379	512	3	105	106	109	116	122	126	132	138	198	203	205	212	224	3	26	88	36	112	119	121	195	196	214	230	252	338	352	387	399	400	432
B/Washington/02/2019	I	T	M	V	E	E	T	P	S	A	A	E	K	K	T	N	C	M	S	K	Y	P	D	E	P	S	S	A	V	N	S	M	F	D	I	V	D	V	I	I	V	K	A	C	V	A	E
B/shandongrencheng/11484/2021	I	A	M	V	E	E	T	L	S	A	A	E	K	E	T	D	C	V	S	K	C	P	N	E	P	S	S	A	V	D	Y	V	F	D	I	V	D	V	I	I	A	K	A	Y	V	A	E
B/shandongrencheng/11485/2021	I	A	M	V	E	E	I	P	C	A	A	D	K	E	N	N	C	V	F	K	C	P	N	E	P	S	S	A	V	N	Y	V	F	D	I	V	D	V	I	I	A	R	A	C	V	A	E
B/shandongrencheng/11486/2021	I	A	M	V	E	E	I	P	S	A	A	D	K	E	N	N	C	V	F	K	C	P	N	E	P	S	S	A	V	N	Y	V	F	D	I	V	D	V	I	I	A	R	A	C	V	A	E
B/shandongrencheng/11487/2021	I	A	I	V	E	E	T	P	S	A	A	E	K	K	T	N	C	V	F	K	C	S	N	E	P	S	S	A	V	N	Y	V	F	D	I	V	D	V	I	I	A	K	A	Y	V	A	E
B/shandongrencheng/11488/2021	I	A	I	V	E	E	T	P	S	A	A	E	K	K	T	N	C	V	S	K	C	P	N	E	P	S	S	A	V	N	Y	V	F	D	I	V	D	V	I	I	A	K	A	Y	V	A	E
B/shandongrencheng/11494/2021	I	A	M	L	E	E	I	P	S	A	A	D	R	E	T	N	C	V	F	K	C	P	N	E	P	S	S	A	I	N	Y	V	F	D	I	V	D	V	I	I	A	R	A	C	V	A	E
B/shandongrencheng/11495/2021	I	A	M	V	E	E	T	P	S	A	A	E	K	E	T	N	C	V	S	E	C	P	N	E	S	S	S	A	V	N	Y	V	F	D	L	V	D	V	I	I	A	K	A	Y	V	A	E
B/shandongrencheng/11499/2021	I	A	I	V	E	E	T	P	S	A	A	E	K	K	T	N	R	V	F	K	C	S	N	D	P	S	S	A	V	N	Y	V	F	D	L	V	G	F	I	I	A	K	A	Y	M	A	E
B/shandongrencheng/11504/2021	I	A	I	V	K	E	T	P	S	A	A	E	K	K	T	N	C	V	S	K	C	P	N	E	P	S	S	A	V	N	Y	V	F	D	I	V	D	V	I	I	A	K	T	Y	V	P	E
B/shandongrencheng/11506/2021	I	A	I	V	E	E	T	P	S	A	A	E	K	K	T	N	C	V	S	K	C	P	N	E	P	S	L	A	V	N	Y	V	F	N	I	V	D	V	I	I	A	K	A	Y	V	A	E
B/shandongrencheng/1115/2022	I	A	M	L	E	E	I	P	S	A	A	D	K	K	T	N	C	V	F	K	C	P	N	E	P	S	S	A	V	N	Y	V	F	D	I	V	D	V	I	I	A	R	A	C	V	A	E
B/shandongrencheng/1122/2022	I	A	M	V	E	E	I	P	S	A	A	D	K	K	N	N	C	V	F	K	C	P	N	E	P	S	S	A	V	N	Y	V	F	D	I	V	D	V	I	I	A	R	A	C	V	A	E
B/shandongrencheng/1125/2022	I	A	I	V	E	E	T	P	S	A	A	E	K	K	T	N	C	V	F	K	C	S	N	E	P	S	S	A	V	N	Y	V	F	D	I	V	D	V	I	I	A	K	A	Y	V	A	E
B/shandongrencheng/1126/2022	I	A	I	V	E	E	T	P	S	A	A	E	K	K	T	N	C	V	S	K	C	P	N	E	P	S	S	A	V	N	Y	V	F	D	I	V	D	V	I	I	A	K	A	Y	V	A	E
B/shandongrencheng/1127/2022	I	A	I	V	E	E	T	P	S	A	A	E	K	K	T	N	C	V	S	K	C	P	N	E	P	S	S	A	V	N	Y	V	F	D	I	V	D	V	I	I	A	K	A	Y	V	A	E
B/shandongrencheng/1128/2022	I	A	M	V	E	E	I	P	S	T	A	D	K	K	T	N	C	V	F	K	C	P	N	E	P	S	S	A	V	N	Y	V	F	D	I	V	D	V	I	I	A	R	A	C	V	A	E
B/shandongrencheng/1169/2022	I	A	M	V	E	E	T	P	S	A	A	E	K	K	T	N	C	V	S	K	C	P	N	E	S	S	S	A	V	N	Y	V	F	D	I	V	D	V	I	I	A	K	A	Y	V	A	E
B/shandongrencheng/1176/2022	I	A	M	L	E	E	T	P	S	A	A	E	K	E	T	N	C	V	S	K	C	P	N	E	S	S	S	V	V	N	Y	V	Y	D	I	V	D	V	I	I	A	K	A	Y	V	A	E
B/shandongrencheng/1210/2022	I	A	M	V	E	E	I	P	S	A	A	D	K	E	T	N	C	V	F	K	C	P	N	E	P	S	S	A	V	N	Y	V	F	D	I	I	D	V	I	I	A	R	A	C	V	A	K
B/shandongrencheng/1211/2022	I	A	I	V	E	E	T	P	S	A	A	E	K	K	T	N	C	V	S	K	C	P	N	E	P	S	S	A	V	N	Y	V	F	D	I	V	D	V	I	I	A	K	A	Y	V	A	E
B/shandongrencheng/1252/2022	T	A	M	L	E	E	T	P	S	A	T	E	K	E	T	D	C	V	S	K	C	P	N	E	P	Y	S	A	V	D	Y	V	F	D	I	V	D	V	V	L	A	K	A	Y	V	A	E
B/shandongrencheng/1291/2022	I	A	I	V	E	E	T	P	S	A	A	E	K	K	T	N	C	V	F	K	C	S	N	E	P	S	S	A	V	N	Y	V	F	D	I	V	D	V	I	I	A	K	A	Y	V	A	E
B/shandongrencheng/1354/2022	I	A	I	V	E	D	T	P	S	A	A	E	K	K	T	N	C	V	S	K	C	P	N	E	P	S	S	A	V	N	Y	V	F	D	I	V	D	V	I	I	A	K	T	Y	V	A	E
B/shandongrencheng/1356/2022	I	A	I	V	E	E	T	P	S	A	A	E	K	K	T	N	C	V	S	K	C	P	N	E	P	S	S	A	V	N	Y	V	F	D	I	V	D	V	I	I	A	K	A	Y	V	A	E
																																															

Virus strain	PA													PB1															PB2																		
	502	504	508	520	522	523	524	528	559	575	670	717	720	48	51	60	184	386	436	454	459	476	566	576	652	686	687	744	14	56	77	109	156	168	181	199	258	268	272	276	313	393	401	468	473	484	590
B/Washington/02/2019	K	Q	R	E	S	S	T	V	V	R	D	L	V	E	N	V	V	R	L	N	M	M	K	K	K	Q	C	A	R	S	I	D	M	I	T	M	S	I	S	S	I	K	I	S	D	N	A
B/shandongrencheng/11484/2021	K	Q	R	E	S	S	T	V	A	G	D	L	V	E	N	V	V	K	I	D	M	M	K	K	K	Q	C	A	R	N	I	N	M	I	T	I	S	I	S	S	T	K	I	S	D	A	A
B/shandongrencheng/11485/2021	K	Q	R	E	S	S	T	V	V	R	D	L	V	D	S	V	V	R	L	D	M	M	K	K	K	Q	C	A	R	N	I	D	M	I	T	I	S	I	S	S	I	K	V	S	D	N	A
B/shandongrencheng/11486/2021	K	Q	R	E	S	S	T	V	V	R	D	L	V	D	S	V	V	R	L	D	M	M	R	K	K	H	D	A	R	N	I	D	M	I	T	I	S	I	S	S	I	K	V	S	D	N	A
B/shandongrencheng/11487/2021	K	Q	R	E	S	S	T	A	V	R	D	L	V	E	N	V	V	R	I	D	M	M	K	K	R	Q	C	T	R	N	I	N	M	I	T	I	S	I	S	S	T	K	I	L	D	N	A
B/shandongrencheng/11488/2021	K	Q	R	E	S	S	T	V	V	R	D	L	V	E	N	V	V	R	I	D	M	M	K	K	K	Q	C	A	K	N	I	N	M	I	T	I	S	I	S	S	I	K	I	S	D	N	A
B/shandongrencheng/11494/2021	K	Q	R	E	S	S	T	V	V	R	D	L	V	D	S	V	V	R	L	D	M	M	K	K	K	Q	C	A	R	N	I	D	M	I	T	I	S	I	S	S	I	E	V	S	D	N	A
B/shandongrencheng/11495/2021	K	Q	R	E	S	S	T	V	A	R	D	L	E	E	N	I	V	R	L	D	M	M	K	K	K	Q	C	A	R	N	I	D	M	I	T	I	S	I	S	S	I	K	I	S	D	N	A
B/shandongrencheng/11499/2021	K	Q	R	E	S	S	T	A	V	R	D	L	V	E	N	V	V	R	I	D	M	K	K	K	R	Q	C	T	R	N	I	N	M	I	T	I	S	I	S	S	T	K	I	L	D	A	A
B/shandongrencheng/11504/2021	K	Q	R	E	S	S	T	V	V	R	D	L	V	E	N	V	V	R	I	D	I	M	K	K	K	Q	C	A	K	N	I	N	V	I	T	I	S	I	S	S	I	K	I	S	D	N	T
B/shandongrencheng/11506/2021	K	Q	R	E	S	S	T	V	V	R	D	L	V	E	N	V	V	R	I	D	M	M	K	K	K	Q	C	A	K	N	I	N	M	K	T	I	S	I	S	S	I	K	I	S	D	N	A
B/shandongrencheng/1115/2022	K	Q	R	E	S	S	T	V	V	R	D	F	V	D	S	V	V	R	L	D	M	M	K	K	K	Q	C	A	R	N	I	D	M	I	T	I	S	I	S	S	I	K	V	S	D	N	A
B/shandongrencheng/1122/2022	K	Q	R	E	S	S	T	V	V	R	D	L	V	D	S	V	V	R	L	D	M	M	K	K	K	Q	C	A	R	N	I	D	M	I	T	I	S	I	S	S	I	K	V	S	N	N	A
B/shandongrencheng/1125/2022	K	Q	R	E	S	S	T	V	V	R	D	L	V	E	N	V	V	R	I	D	M	K	K	K	R	Q	C	T	R	N	I	N	M	I	T	I	S	I	S	S	T	K	I	L	D	A	A
B/shandongrencheng/1126/2022	K	Q	R	E	S	S	T	V	V	R	D	L	V	E	N	V	V	R	I	D	M	M	K	K	K	Q	C	A	K	N	I	N	M	I	T	I	S	I	S	S	I	K	I	S	D	N	A
B/shandongrencheng/1127/2022	K	Q	R	E	S	S	T	V	V	R	D	L	V	E	N	V	V	R	L	D	M	M	K	K	K	Q	C	A	K	N	I	N	M	I	T	I	S	I	S	S	I	K	I	S	D	N	A
B/shandongrencheng/1128/2022	K	Q	R	E	S	S	T	V	V	R	E	L	V	D	S	V	V	R	L	D	M	M	K	K	K	Q	C	A	R	N	I	D	M	I	T	I	S	I	S	S	I	K	V	S	D	A	A
B/shandongrencheng/1169/2022	K	Q	R	E	S	S	T	V	V	R	D	L	V	E	N	I	V	R	L	D	M	M	K	K	K	Q	C	A	R	N	M	N	M	I	T	I	T	K	A	T	I	K	I	S	D	N	A
B/shandongrencheng/1176/2022	K	Q	R	E	S	S	T	V	V	R	D	L	V	E	N	I	I	R	L	D	M	M	K	K	K	Q	C	A	R	N	I	N	M	I	T	I	S	I	S	S	T	K	I	S	D	N	A
B/shandongrencheng/1210/2022	K	Q	R	E	S	S	T	V	V	R	D	L	V	D	S	V	V	R	L	D	M	M	K	K	K	Q	C	A	R	N	I	D	M	I	T	I	S	I	S	S	I	K	V	S	D	A	A
B/shandongrencheng/1211/2022	K	Q	R	E	S	S	T	V	V	R	D	L	V	E	N	V	V	R	I	D	M	M	K	K	K	Q	C	A	K	N	I	N	M	I	T	I	S	I	S	S	I	K	I	S	D	N	A
B/shandongrencheng/1252/2022	K	Q	R	E	S	S	T	V	V	R	D	L	V	E	N	V	V	R	I	D	M	M	K	R	K	Q	C	A	R	N	I	N	M	I	T	I	S	I	S	S	I	K	I	S	D	N	A
B/shandongrencheng/1291/2022	E	R	G	G	G	G	A	A	V	R	D	L	V	E	N	V	V	R	I	D	M	K	K	K	R	Q	C	T	R	N	I	N	M	I	T	I	S	I	S	S	T	K	I	S	D	N	A
B/shandongrencheng/1354/2022	K	Q	R	E	S	S	T	V	V	R	D	L	V	E	N	V	V	R	I	D	M	M	K	K	K	Q	C	A	K	N	I	N	M	I	T	I	S	I	S	S	I	K	I	S	D	A	T
B/shandongrencheng/1356/2022	K	Q	R	E	S	S	T	V	V	R	D	L	V	E	N	V	V	R	I	D	M	M	K	K	K	Q	C	A	K	N	I	N	M	I	T	I	S	I	S	S	I	K	I	S	D	N	A

The active catalytic site of NA protein is composed of 19 amino acids, including R116, E117, D149, R150, R154, W177, S178, D197, I221, R223, E226, H273, E275, E276, R292, N294, R374, Y409, and E428 (Chen et al., 2019). Among the 24 sequences, 20 sites of NA protein were mutated, and one of the sequences had the D197N mutation in the catalytic site of NA. The NA inhibitors are the primary antiviral drugs used to treat influenza. However, mutations such as E105K, P139S, G140R, D197N, and H273Y in BV influenza viruses can cause resistance to NA inhibitors (Farrukee et al., 2015, 2018). In this study, it was observed that one sequence had a D197N drug resistance mutation.

Nucleoprotein is a crucial structural protein that makes up the nucleocapsid of the influenza virus. It contains specific amino acid sites that are important for its nuclear import and export, including K44, R45, and F209, and the 125–149 aa region of the RNA-binding domain of NP (Ng et al., 2012; Sherry et al., 2014). In the 24 sequences analyzed, nine amino acid sites in NP proteins were found to be mutated, with the E128D mutation appearing in the RNA binding domain of 7 sequences.

M1 is a crucial matrix protein that forms the structure of the influenza virus. It contains a nuclear localization signal (NLS), nuclear export signal (NES), and phosphorylation modification sites, which play a role in promoting the nuclear export of influenza virus ribonucleoprotein (vRNP). In the influenza B virus, the NLS is located at amino acids 76–94, while the NES is located at amino acids 3–14 and 124–133. The key phosphorylation sites are T80 and S84 (Cao et al., 2014). The present study found that none of the 24 sequences analyzed showed mutations in the NLS, NES, T80, and S84.

NS1 and NEP are nonstructural proteins of influenza; NS1 has diverse biological functions, including binding to host mRNA, inhibiting interferon, and interacting with many host proteins (Nogales et al., 2018). It consists of two functional domains, an N-terminal RNA-binding domain (RBD, 1–90 aa) and a C-terminal effector domain (ED, 120–261 aa), which are connected by a short interdomain linker region (LR, 91–119 aa) (Jumat et al., 2016). Among the 24 sequences analyzed, 14 amino acid site variations were observed in NS1, with 1 variation found in the RBD, 4in the linker region, and 9 in the effector domain. On the contrary, NEP is a nuclear export protein that facilitates the nuclear export of viral RNP. Its nuclear export signal (NES) is located at 11–23 aa of NEP (Paragas et al., 2001). In the 24 sequences, three amino acids were identified in NEP, but none of them were found in the NES sites.

Polymerase acidic inhibitors are currently important specific drugs for treating influenza B, and their sites of action are T20, F24, M34, N37, and I38. None of the 24 strains examined in this study had mutations at these sites (Takashita, 2021). However, the K338R mutation in PA was present in 7 out of 24 sequences and is known to enhance the replication ability of influenza (Bae et al., 2018). The PB1 gene can also undergo mutations that enhance replication ability (Binh et al., 2013), such as D27V/N and N44Q; however, none of the 24 sequences had mutations in these sites. PB2 binds to the cap structure through Q325, W359, and Y434 alleles (Kim et al., 2018), and none of the 24 sequences had mutations in these sites either.

In this study, multiple amino acid variations were observed in the PA, HA, NA, NP, and NS1 proteins of the influenza B virus, with some mutations potentially affecting drug resistance and viral replication. In contrast, the M1 and NEP proteins showed no mutations in the analyzed sequences.

### 3.5. Glycosylation site analysis

Glycosylation of the HA and NA of influenza virus is a crucial mechanism for immune evasion and persistent viral infection (Kim et al., 2018). One HA sequence has an N59K mutation that eliminates a glycosylation site in N59. The N197D variant in all 24 sequences results in the loss of the glycosylation site at N197. An S109F mutation in NS1 of 11 sequences also leads to losing a glycosylation site at N107. Finally, one PB2 sequence has a D473N mutation, creating a new glycosylation site at N473 (Table 3). Overall, the mutations N59K in HA, N197D in both HA and NA, S109F in NS1, and D473N in PB2 alter glycosylation sites, potentially affecting immune evasion and persistent viral infection.

## 4. Discussion

Over the past two decades, studies on viruses have been extensively carried out due to their association with both acute self-limiting and long-term chronic diseases in humans, including the influenza virus responsible for the common “flu” (Li et al., 2016; Chen X. et al., 2017; Chen Y. et al., 2017; Jiao et al., 2017; Shi et al., 2017; Duan et al., 2018; Yuan et al., 2023). Many pathogens cause influenza-like illnesses, the most important of which is the influenza virus (Clementi et al., 2021). Influenza virus infection can cause severe disease burden, causing fever, cough, headache, pneumonia, and even death, resulting in a severe disease burden (Feng et al., 2020; Castillo-Rodriguez et al., 2022). The WHO influenza established a global surveillance network and recommends annual influenza vaccine strains for the northern and southern hemispheres. China is one of the most crucial member countries for influenza surveillance and has established a surveillance network covering all prefecture-level cities (Liu et al., 2022). Jining City is an ordinary medium-sized prefecture-level city in northern China, with a total area of 11,000 km^2^ and a population of 8.358 million. It has a warm temperate monsoon climate with four distinct seasons. Jining City has built an influenza surveillance network laboratory. According to the epidemic characteristics of influenza with high incidence in winter and spring, about 1,500 influenza-like case specimens from two sentinel hospitals are monitored for influenza from April to March of the following year.

From April 2021 to March 2022, Jining City’s influenza surveillance network laboratory reported a 24.63% positive rate of influenza virus among influenza-like illnesses, with BV influenza virus being the primary prevalent subtype during the winter and spring seasons in northern China (Feng et al., 2020; Liu et al., 2022). The highest positive rate was observed in December and January, followed by November and February, while the lowest positive rate was observed from April to September. Additionally, the percentage distributions by age category showed slightly higher rates in the 6–60 year groups and lower rates in the 0–5 year groups and above 65 year groups as shown in Table 1.

Compared to the vaccine strain B/Washington/02/2019, the HA and PB2 gene segments of the Jining strain exhibit the lowest similarity and the highest gene evolution distance. Although both strains belong to the Victoria clade 1A branch on the evolutionary tree (162–164 aa missing in HA); they are located in different clades. Jining has two epidemic strains the Victoria clade 1A.3a.1 and Victoria clade 1A.3a.2, which are cocirculating. In the 2019–2020 influenza season, BV lineage influenza viruses in Jining City were mainly located in the Victoria clade 1A.3 branch, indicating that the BV lineage influenza viruses in Jining City have evolved from the Victoria clade 1A.3 branch to the Victoria clade 1A.3a.1 and Victoria clade 1A.3a.2 evolutionary branches. In addition, on the phylogenetic tree, the isolated strains worldwide are also mainly distributed in the Victoria clade 1A.3a.1 and Victoria clade 1A.3a.2 evolutionary branches. However, a small number of strains in the 2021–2022 season were located in the Victoria clade 1A.3 branch, indicating that the global circulation of BV lineage influenza viruses in the 2021–2022 season is a polymorphic epidemic with multiple units coexisting, mainly in the Victoria clade 1A.3a.1 and Victoria clade 1A.3a.2 evolutionary branches.

Variant strains with different amino acids on hemagglutinin (HA) continue to emerge due to the high mutation rate of influenza viruses. When there are more than four amino acid variations in the HA antigenic determinants, and the variations are distributed on at least two antigenic determinants, antigenic drift occurs, forming a new influenza variant (Liu et al., 2018). Out of the 24 sequences analyzed, the HA epitope of 7 sequences located in the Victoria clade 1A.3a.1 only had 3 amino acid changes. In contrast, the HA epitope of 17 sequences found in the Victoria clade 1A.3a.2 clusters had 5–8 amino acid site variations distributed across 3 or 4 antigenic determinants. These variations may change the antigenicity of the influenza virus, lead to the formation of new variants, and possibly affect the protective effect of vaccine strains. The receptor binding sites of HA have 2–3 amino acid mutations, which may impact the binding of HA and receptors. The N197D variant found in all 24 sequences is not only located in the antigenic epitope of HA but also in the receptor binding site. However, the mutation of N197, an important glycosylation site in the B/Victoria virus, resulted in the loss of the N197 glycosylation site in these 24 sequences. This loss, in turn, affects the changes in HA antigenicity and promotes the reproduction of the influenza virus in embryonated eggs (Nakagawa et al., 2004; Saito et al., 2004). Currently, NA, PA, PB1, and PB2 inhibitors are all specific drugs used for the treatment of influenza A virus. However, only NA and PA inhibitors are specific drugs for the treatment of influenza B infection (Takashita, 2021). The function of NA is to cleave the glycosidic bonds between the HA and the influenza receptor, allowing the virus to be released from the host cell surface. The NA inhibitors can specifically bind to the active site of the NA enzyme, inhibit the activity of the NA enzyme, and inhibit the release of the virus. PA is a component of the polymerase of the influenza virus, and PA inhibitors can inhibit the endonuclease activity of the viral polymerase, thereby inhibiting the replication of the influenza virus. However, with the continuous mutation of the influenza virus genes, especially when the active site or adjacent amino acid sites of NA and PA undergo mutations, it may reduce the binding of the inhibitor to NA and PA, and reduce the virus’s sensitivity to the inhibitor, resulting in drug-resistant mutants that make the treatment of influenza more challenging (Takashita, 2021). The D197N mutation may result in resistance to NA inhibitors, indicating the possible emergence of NA inhibitor-resistant variants in B/Victoria influenza viruses in Jining City. Studies have shown that the N197D mutation can affect the antigenic properties of the HA protein, potentially allowing the virus to evade recognition by the host immune system. This is because the glycosylation site can act as a shield to mask the virus from antibodies produced by the host. Additionally, the mutation may alter the shape of the HA protein, making it more difficult for antibodies to bind and neutralize the virus (Tsai and Tsai, 2019). This demonstrates that PA and NA inhibitors can still be used to treat influenza, but there is a need to strengthen the monitoring of drug-resistant mutations.

PA, PB1, and PB2 are the three subunits that make up the RNA polymerase of influenza virus (Wandzik et al., 2021). Among them, K338 of PA is located in the polymerase’s core position. The K338R mutation in PA has been shown to enhance the activity of B/Victoria influenza virus RNA polymerase, thereby increasing its pathogenicity (Bae et al., 2018). In this study, it was found that none of the 17 sequences in the Victoria clade 1A.3a.2 had the K338R mutation, while all 7 sequences in the Victoria clade 1A.3a.1 had the K338R mutation in their PA subunit.

This study analyzed the influenza epidemic and gene evolution variation in Jining City from 2021 to 2022, showing that the B/Victoria influenza virus’s antigenic epitopes have partially mutated and formed new variants. These new variants are poorly matched with the WHO-recommended northern hemisphere vaccine strains, which should be adjusted accordingly. Furthermore, the 24 nucleic acid and protein sequences in Jining have undergone some variation, displaying differences in variation sites, homology, evolutionary characteristics, and genetic distances. This suggests that the B/Victoria strain of influenza virus is still evolving and mutating; thus, influenza surveillance needs further strengthening.

## Data availability statement

The datasets presented in this study can be found in online repositories. The names of the repository/repositories and accession number(s) can be found in the article/Supplementary material.

## Ethics statement

The studies involving human participants were reviewed and approved by Ethics Committee at Jining Center for Disease Control and Prevention. Written informed consent to participate in this study was provided by the participants’ legal guardian/next of kin.

## Author contributions

LL, TL, and BJ conceived and designed the experiments. LL performed the experiments and analyzed the data. LL, QW, YD, TL, YaJ, ZW, XW, HD, YoJ, and BJ interpreted the data. LL, QW, and BJ wrote the manuscript. All authors contributed to the article and approved the submitted version.

## Funding

This study was supported by Science and Technology Development Funds for Shandong Medical and Health (202112060725) and Key Research and Development Funds for Jining Medical and Health (2021018).

## Conflict of interest

ZW was employed by Nanjing Chengshi BioTech (TheraRNA) Co., Ltd.

The remaining authors declare that the research was conducted in the absence of any commercial or financial relationships that could be construed as a potential conflict of interest.

## Publisher’s note

All claims expressed in this article are solely those of the authors and do not necessarily represent those of their affiliated organizations, or those of the publisher, the editors and the reviewers. Any product that may be evaluated in this article, or claim that may be made by its manufacturer, is not guaranteed or endorsed by the publisher.

## Supplementary material

The Supplementary material for this article can be found online at: https://www.frontiersin.org/articles/10.3389/fmicb.2023.1196451/full#supplementary-material

## Footnotes

^1^ www.gisaid.org
