# Supplementary material for: Genetic characterization and whole-genome sequencing-based genetic analysis of influenza virus in Jining City during 2021–2022
Source: Front Microbiol. 2023 Jun 22;14:1196451. doi: 10.3389/fmicb.2023.1196451 (PMC10324579; doi:10.3389/fmicb.2023.1196451)
Supplement: Supplementary file 1 [file Table_1.pdf]

Supplemental Table 1. GISAID Isolate ID

Name	GISAID ID
B/shandongrencheng/11484/2021	EPI_ISL_17527251
B/shandongrencheng/11485/2021	EPI_ISL_17527252
B/shandongrencheng/11486/2021	EPI_ISL_17527256
B/shandongrencheng/11487/2021	EPI_ISL_17527255
B/shandongrencheng/11488/2021	EPI_ISL_17527257
B/shandongrencheng/11494/2021	EPI_ISL_17527258
B/shandongrencheng/11495/2021	EPI_ISL_17527259
B/shandongrencheng/11499/2021	EPI_ISL_17527260
B/shandongrencheng/11504/2021	EPI_ISL_17527261
B/shandongrencheng/11506/2021	EPI_ISL_17527273
B/shandongrencheng/1115/2022	EPI_ISL_17526593
B/shandongrencheng/1122/2022	EPI_ISL_17526592
B/shandongrencheng/1125/2022	EPI_ISL_17526594
B/shandongrencheng/1126/2022	EPI_ISL_17526595
B/shandongrencheng/1127/2022	EPI_ISL_17526596
B/shandongrencheng/1128/2022	EPI_ISL_17526597
B/shandongrencheng/1169/2022	EPI_ISL_17526598
B/shandongrencheng/1176/2022	EPI_ISL_17526599
B/shandongrencheng/1210/2022	EPI_ISL_17526600
B/shandongrencheng/1211/2022	EPI_ISL_17527246
B/shandongrencheng/1252/2022	EPI_ISL_17527247
B/shandongrencheng/1291/2022	EPI_ISL_17527248
B/shandongrencheng/1354/2022	EPI_ISL_17527249
B/shandongrencheng/1356/2022	EPI_ISL_17527250
